# Supplementary material for: Effect of Single High Dose Vitamin D Substitution in Hospitalized COVID-19 Patients with Vitamin D Deficiency on Length of Hospital Stay
Source: Biomedicines. 2023 Apr 25;11(5):1277. doi: 10.3390/biomedicines11051277 (PMC10215464; doi:10.3390/biomedicines11051277)
Supplement: Supplementary file 1 [file biomedicines-11-01277-s001.zip › biomedicines-2298831-supplementary.docx]

Supplementary Material

Effect of Single High Dose Vitamin D Substitution in Hospitalized COVID-19 Patients with Vitamin D Deficiency on Length of Hospital Stay.

**Supplementary Table S1**. Visit procedure and collected information for each patient at different time points during the study.

|  | **Screening** | **Baseline ^a^** | **Treatment Phase (Daily)** | | | **Discharge or fatality** | | **Baseline**  **+ 28 d** | | **Baseline**  **+ 90 d** |
| --- | --- | --- | --- | --- | --- | --- | --- | --- | --- | --- |
| Eligibility Criteria | X | X |  | | |  | |  | |  |
| Informed Consent | X | | |  |  | |  | |  | |
| Demographic Information |  | X |  | | |  | |  | |  |
| Smoking status |  | X |  | | |  | |  | |  |
| Height and weight |  | X |  | | |  | |  | |  |
| Vaccination status (Influenza) |  | X |  | | |  | |  | |  |
| Covid-19 symptoms |  | X |  | | |  | |  | |  |
| PCR Testing history |  | X |  | | |  | |  | |  |
| Chest imaging history |  | X |  | | |  | |  | |  |
| Assessment of Co-Morbidities |  | X |  | | |  | |  | |  |
| Hospitalisation date (admission/discharge) |  | X |  | | | X | |  | |  |
| Course of the hospitalization ^b^ |  |  | X | | | X | |  | |  |
| Discharge management ^c^ |  |  |  | | | X | |  | |  |
| Routine laboratory assessment ^d^ |  | X | X | | | X | |  | |  |
| Additional laboratory examination ^e^ |  | (X) |  | | | (X) | |  | |  |
| Health related quality of Life (SF-12) |  | X |  | | |  | | X | | X |
| Concomitant medication |  | X | X | | | X | |  | |  |
| Symptom Diary |  | X | X | | | X | |  | |  |
| Blood pressure |  | X | X | | | X | |  | |  |
| Heart rate |  | X | X | | | X | |  | |  |
| O_2_ saturation |  | X | X | | | X | |  | |  |
| Temperature |  | X | X | | | X | |  | |  |
| Respiratory rate |  | X | X | | | X | |  | |  |
| Oxygen requirement |  | X | X | | | X | |  | |  |
| High dose vitamin D_3_ or placebo (Intervention) |  | X |  | | |  | |  | |  |
| 800IU vitamin D_3_ (Treatment as usual) |  |  | X | | | X | |  | |  |
| ^a^ The baseline visit corresponds to the day of randomization  ^b^ Course of the hospitalization: it was assessed whether patients were admitted to intensive care, if yes for how long and if they were mechanically ventilated, including other complications due to Covid-19.  ^c^ With discharge management it was assessed where the participants were discharged to and how the aftercare was organized.  ^d^ Routine laboratory assessment had to be done at least every seven days and at discharge.  ^e^ Additional laboratory values, are only taken if they were not taken routinely and include the following parameters: 25-hydroxyvitamin D, Calcium and PTH. | | | | | | | | | | |

**Supplementary Table S2**. Co-Morbidities at time of randomization.

| **Co-Morbidities** | **N  (Intervention/Control)^a^** | **Intervention**  **% (n)** | **Control**  **% (n)** | **p-value** |
| --- | --- | --- | --- | --- |
| None | 39/39 | 35.9 (14) | 33.3 (13) | 1.00‡ |
| Arterial hypertension | 39/39 | 20.5 (8) | 33.3 (13) | .307‡ |
| Cardiovascular disease | 39/39 | 20.5 (8) | 25.6 (10) | .789‡ |
| Diabetes mellitus | 39/39 | 23.1 (9) | 20.5 (8) | 1.00‡ |
| Chronic respiratory disease ^b^ | 39/39 | 17.9 (7) | 17.9 (7) | 1.00 ‡ |
| Renal disease | 39/39 | 5.1 (2) | 5.1 (2) | 1.00‡ |
| Immunologic disease | 39/39 | 2.6 (1) | 2.6 (1) | 1.00‡ |
| Liver disease | 39/39 | 0 (0) | 5.1 (2) | .494‡ |
| Allergies | 39/39 | 2.6 (1) | 0 (0) | 1.00‡ |
| Cancer ^c^ | 39/39 | 0 (0) | 2.6 (1) | .314 ‡ |
| Other | 39/39 | 33.3 (13) | 33.3 (13) | 1.00‡ |
| Categorical variables: percentages of patients (absolute number of patients per group = n).  ‡ Fisher’s exact test when expected frequencies <5 instead of Chi2 test for categorical variables  a number of patients with available information  b Chronic lung disease: including Asthma and COPD, no other conditions were reported  c active malignancy was an exclusion criterion  level of significance: * < .05, ** < .01 *** < .001 | | | | |

**Supplementary Table S3**. Concomitant medication during hospitalization.

| **Medication** | **N (Intervention/Control)^a^** | **Intervention**  **% (n)** | **Control**  **% (n)** | **P-value** |
| --- | --- | --- | --- | --- |
| Anti-thrombotic treatment | 39/39 | 97.4 (38) | 100 (39) | 1.00‡ |
| Antibiotic treatment | 39/39 | 41.0 (16) | 51.3 (20) | .496‡ |
| **Systemic Steroids** | 39/39 |  |  |  |
| Dexamethasone |  | 82.1 (32) | 82.1 (32) | 1.00‡ |
| Prednisolone |  | 5.1 (2) | 5.1 (2) | 1.00‡ |
| NSAID ^b^ | 39/39 | 38.5 (15) | 30.8 (12) | .635‡ |
| Inhaler ^b^ | 39/39 | 33.3 (13) | 43.6 (17) | .485‡ |
| Remdesivir |  | 10.3 (4) | 23.1 (9) | .224‡ |
| Nirmatrelvir/Ritonavir |  | 0 (0) | 0 (0) | - |
| Tocilizumab | 39/39 | 7.7 (3) | 5.1 (2) | 1.00‡ |
| Other medication | 39/39 | 87.2 (34) | 92.3 (36) | .711‡ |
| Categorical variables: percentages of patients (absolute number of patients per group = n).  ‡ Fisher’s exact test when expected frequencies <5 instead of Chi^2^ test for categorical variables  ^a^ number of patients with available information.  ^b^ Inhaler included: Inhaled corticosteroids (ICS), long acting β- 2 agonist (LABA), or a combination of those (ICS+LABA), long-acting muscarinic antagonists (LAMA), LAMA+ LABA combinations and short acting β-2 agonists (SABA).  level of significance: * < .05, ** < .01 *** < .001 | | | | |

**Supplementary Table S4**. Robust linear regression results presenting the effect of group. Intervention vs. Control on time to discharge (from randomization) (univariable and adjusted for COVID-19 risk factors, prognostic imbalances, and center effects.

| **time from randomization to discharge** | Beta | **95% confidence interval** | |
| --- | --- | --- | --- |
|  |  | Lower Level | Upper Level |
| (Intercept) | 6.78 | 5.33 | 8.24 |
| Group | 0.44 | -1.62 | 2.50 |
| **Adjusted for risk factors ^a^** |  |  |  |
| (Intercept) | 7.10 | 4.14 | 10.06 |
| Group | 0.03 | -2.17 | 2.22 |
| **Adjusted for O2, dyspnea, flu like symptoms ^b^** |  |  |  |
| (Intercept) | 6.25 | 4.16 | 8.35 |
| Group | 0.39 | -1.78 | 2.56 |
| **Adjusted for center** |  |  |  |
| (Intercept) | 6.63 | 5.14 | 8.12 |
| Group | 0.74 | -1.25 | 2.73 |
| Beta = Average change in LOS between the groups (intervention vs. control group)  ^a^ Adjusted for risk factors age, the number of comorbidities, SpO2, the number of symptoms at inclusion, and 25(OH)D level at randomization.  ^b^ Adjusted for (p<0.10): O2, dyspnea, flu like symptoms. | | | |

**Supplementary Table S5**. Mean SF-12 mental and SF-12 physical scores at randomization, 28 days and 90 days after randomization.

| **Health related quality of life** | **N  (Intervention/Control)^a^** | **Intervention** | **Control** | **p-value** |
| --- | --- | --- | --- | --- |
| **Mental Score** |  |  |  |  |
| SF-12 Mental Score at randomization | 26/20 | 41.76 ± 15.510  [6.36 – 66.66] | 51.66 ± 8.044  [29.69 – 61.09] | **.018†** |
| 28-days follow-up SF-12 Mental Score | 19/17 | 51.09 ± 13.57 [23.25 – 66.28] | 51.86 ± 8.82 [33.00 – 62.13] | .775† |
| 90 days follow-up SF-12 Mental Score | 19/14 | 47.58 ± 11.79 [17.10 – 60.76] | 52.81 ± 9.46 [28.68 – 63.17] | .126† |
| **Physical Score** |  |  |  |  |
| SF-12 Physical Score at randomization | 26/20 | 31.39 ± 10.61 [15.80 – 56.58] | 27.85 ± 11.75 [10.55 – 49.78] | .352† |
| 28-days follow-up SF-12 Physical Score | 19/17 | 38.73 ± 9.14  [27.32 – 56.58] | 43.41 ± 10.35 [25.74 – 56.58] | .136† |
| 90-days follow-up SF-12 Physical Score | 19/14 | 45.07 ± 11.72 [23.39 – 61.53] | 50.81 ± 5.70 [39.70 – 58.23] | .259† |
| Continuous variables: mean ± standard deviation (SD) [range].  ^a^ number of patients with available information  † Man-Whitney-U-Test  level of significance: * < .05, ** < .01 *** < .001 | | | | |

**Supplementary Table S6**. Mixed-effects linear regression results on SF-12 mental and physical outcome with a time and group interaction term.

| **Treatment by Time Interaction** | **Estimate** | **95% CI lower** | **95% CI upper** | **df** | **p-value** |
| --- | --- | --- | --- | --- | --- |
| **SF-12 mental** |  |  |  |  |  |
| (Intercept) | 51.55 | 46.63 | 56.48 | 102.6 | **<.001***** |
| Group | -8.13 | -14.70 | -1.56 | 102.9 | **0.016*** |
| Time | 0.35 | -3.37 | 4.10 | 82.1 | 0.852 |
| Group* time | 2.75 | -2.19 | 7.69 | 82.1 | 0.272 |
| **SF-12 physical** |  |  |  |  |  |
| (Intercept) | 29.04 | 46.63 | 56.48 | 89.7 | **< .001***** |
| Group | 2.38 | -14.70 | -1.56 | 90.1 | 0.409 |
| Time | 11.80 | -3.37 | 4.10 | 77.4 | **< 0.001***** |
| Group* time | -4.56 | -2.19 | 7.69 | 77.5 | **0.023*** |
| *Abbreviations: df= degrees of freedom*  *p-value based on Satterthwaite approximation*  *level of significance: * < .05, ** < .01 *** < .001* | | | | | |

**Supplementary Table S7**. Frequency of self- reported symptoms at day 5 after randomization.

| **Symptoms on day 5 after randomization** | **N  (Intervention/Control)^a^** | **Intervention** | **Control** | **P-value** |
| --- | --- | --- | --- | --- |
| Cough | 16/20 | 81.3 (13) | 70 (14) | .700‡ |
| Breathlessness | 16/20 | 43.8 (7) | 25 (5) | .298‡ |
| Need for Oxygen | 23/34 | 30.4 (7) | 44.1 (15) | .407‡ |
| Fatigue | 24/34 | 8.3 (2) | 5.9 (2) | 1.00‡ |
| Loss of Taste | 16/17 | 25.1 (4) | 17.6 (3) | .570 |
| Vertigo | 24/34 | 8.3 (2) | 0 (0) | .167‡ |
| Headache | 16/20 | 56.3 (9) | 15 (3) | .014‡* |
| GI Symptoms | 24/34 | 4.2 (1) | 8.8 (3) | .635‡ |
| Neurological Symptoms | 24/34 | 0 (0) | 2.9 (1) | 1.00‡ |
| Flu Like Symptoms | 23/34 | 0 (0) | 2.9 (1) | 1.00‡ |
| Chest Pain | 16/20 | 31.3 (5) | 10 (2) | .204‡ |
| Other | 24/34 | 8.3 (2) | 2.9 (1) | .564‡ |
| Categorical variables: percentages of patients (absolute number of patients per group = n).  ^a^ number of patients with available information  Flu like symptoms: include chills, sore throat, runny nose, sinusitis, joint pain, sneezing  Gastrointestinal (GI) Symptoms: include nausea, vomiting, diarrhea, obstipation and abdominal pain or loss of appetite  Neurological Symptoms: include balance disturbance, impaired vision, impaired concentration, myalgia, weakness  ‡ p- value based on Fisher’s exact test when expected frequencies <5 instead of Chi^2^ test for categorical variables  level of significance: * < .05, ** < .01 *** < .001 | | | | |

**Supplementary Table S8**. Mean vital signs at day 5 after randomization.

| **Vital Signs on day 5 after randomization** | | **N**  **(Intervention/Control)^a^** | **Intervention** | **Control** | **p-value** |
| --- | --- | --- | --- | --- | --- |
| BP Systolic | mmHg | 22/34 | 126 ± 14 | 131 ± 21 | .300 |
| BP Diastolic | mmHg | 22/34 | 75 ± 10 | 74 ± 8 | .534 |
| Pulse | bpm | 23/34 | 76 ± 13 | 75 ± 14 | .746 |
| Temperature | °C | 23/34 | 37.0 ± .738 | 36.9 ± .696 | .607† |
| Respiratory Rate | /min | 19/29 | 20 ± 5 | 21 ± 5 | .460† |
| SpO_2_ | % | 23/34 | 92 ± 2.5 | 93 ± 2.5 | .311† |
| Abbreviations: BP = Blood Pressure, SpO_2_= peripheral oxygen saturation  Continuous variables: mean ± standard deviation (SD)  ^a^ number of patients with available information  † Man-Whitney-U-Test  level of significance: * < .05, ** < .01 *** < .001 | | | | | |

**Supplementary Table S9**. Frequency of self- reported symptoms at day 10 after randomization.

| **Symptoms on day 10** | **N  (Intervention/Control)^a^** | **Intervention** | **Control** | **p-value** |
| --- | --- | --- | --- | --- |
| Cough | 3/5 | 33.3 (1) | 80 (4) | .464‡ |
| Breathlessness | 3/5 | 66.6 (2) | 49 (2) | 1.00‡ |
| Need for Oxygen | 10/10 | 50 (5) | 50 (5) | 1.00‡ |
| Fatigue | 10/11 | 0 (0) | 0 (0) | - |
| Loss of Taste | 3/5 | 0 (0) | 0 (0) | - |
| Vertigo | 10/ 11 | 10 (1) | 0 (0) | .476‡ |
| Headache | 3/5 | 66.7(2) | 20 (1) | .464**‡** |
| GI Symptoms | 10/11 | 0 (0) | 0 (0) | - |
| Neurological Symptoms | 10/11 | 0 (0) | 0 (0) | - |
| Flu Like Symptoms | 10/11 | 0 (0) | 0 (0) | - |
| Chest Pain | 3/5 | 66.7(2) | 20 (1) | .464‡ |
| Other | 10/11 | 10 (1) | 9.1 (1) | 1.00 ‡ |
| Categorical variables: percentages of patients (absolute number of patients per group = n).  ^a^ number of patients with available information  Flu like symptoms: include chills, sore throat, runny nose, sinusitis, joint pain, sneezing  Gastrointestinal (GI) Symptoms: include nausea, vomiting, diarrhea, obstipation and abdominal pain or loss of appetite  Neurological Symptoms: include balance disturbance, impaired vision, impaired concentration, myalgia, weakness  ‡ p- value based on Fisher’s exact test when expected frequencies <5 instead of Chi^2^ test for categorical variables  level of significance: * < .05, ** < .01 *** < .001 | | | | |

**Supplementary Table S10**. Mean vital signs at day 10 after randomization.

| **Vital Signs on day 10 after randomization** | | **N**  **(Intervention/Control) ^a^** | **Intervention** | **Control** | **p-value** |
| --- | --- | --- | --- | --- | --- |
| BP Systolic | mmHg | 10/11 | 113 ± 10 | 131 ± 22 | .029* |
| BP Diastolic | mmHg | 10/11 | 73 ± 11 | 71 ± 10 | .666 |
| Pulse | bpm | 10/11 | 71±11 | 75 ± 12 | .684 |
| Temperature | °C | 10/11 | 36.8 ± .259 | 36.7± .467 | .836 |
| Respiratory Rate | /min | 10/11 | 20 ± 5 | 22 ± 6 | .182 |
| SpO_2_ | % | 10/11 | 92 ± 2.5 | 92 ± 3 | .705† |
| Abbreviations: BP = Blood Pressure, SpO_2_= peripheral oxygen saturation  Continuous variables: mean ± standard deviation (SD)  ^a^ number of patients with available information  † Man-Whitney-U-Test  level of significance: * < .05, ** < .01 *** < .001 | | | | | |

**Supplementary Table S11**. Frequency of adverse and serious adverse events occurred during the trial per study group.

| **Safety events** | **N  (Intervention/Control)** | **Intervention** | **Control** | **p-value** |
| --- | --- | --- | --- | --- |
| Adverse Events | 39/39 | 10.3 (4) | 25.6 (10) | .138‡ |
| Serious Adverse Events | 39/39 | 10.3 (4) | 5.1 (2) | .675‡ |
| Categorical variables: percentages of patients (absolute number of patients per group = n).  † Man-Whitney-U-Test  level of significance: * < .05, ** < .01 *** < .001 | | | | |

**Supplementary Table S12**. Levels of calcium, PTH and phosphorus at time of discharge.

|  | **N available (Intervention/Control)^a^** | **Intervention** | **Control** | **p-value** |
| --- | --- | --- | --- | --- |
| Calcium level at discharge in mmol/l | 16/21 | 2.30 ± .099  [2.21 – 2.46] | 2.22 ± .063  [2.14 – 2.30] | .101 |
| PTH level at discharge in pmol/l | 15/21 | 2.98 ± 1.30  [1.35 –5.77] | 4.63 ± 2.16  [1.60 – 10.0] | **.007**** |
| Phosphate level at discharge in mmol/l | 7/12 | 1.01 ± .289  [.55 – 1.37] | .86 ± .266  [.400 – 1.36] | **.003**** |
| *Continuous variables: mean ± standard deviation (SD) [range]*  *^a^ number of patients with available information*  *level of significance: * < .05, ** < .01 *** < .001* | | | | |


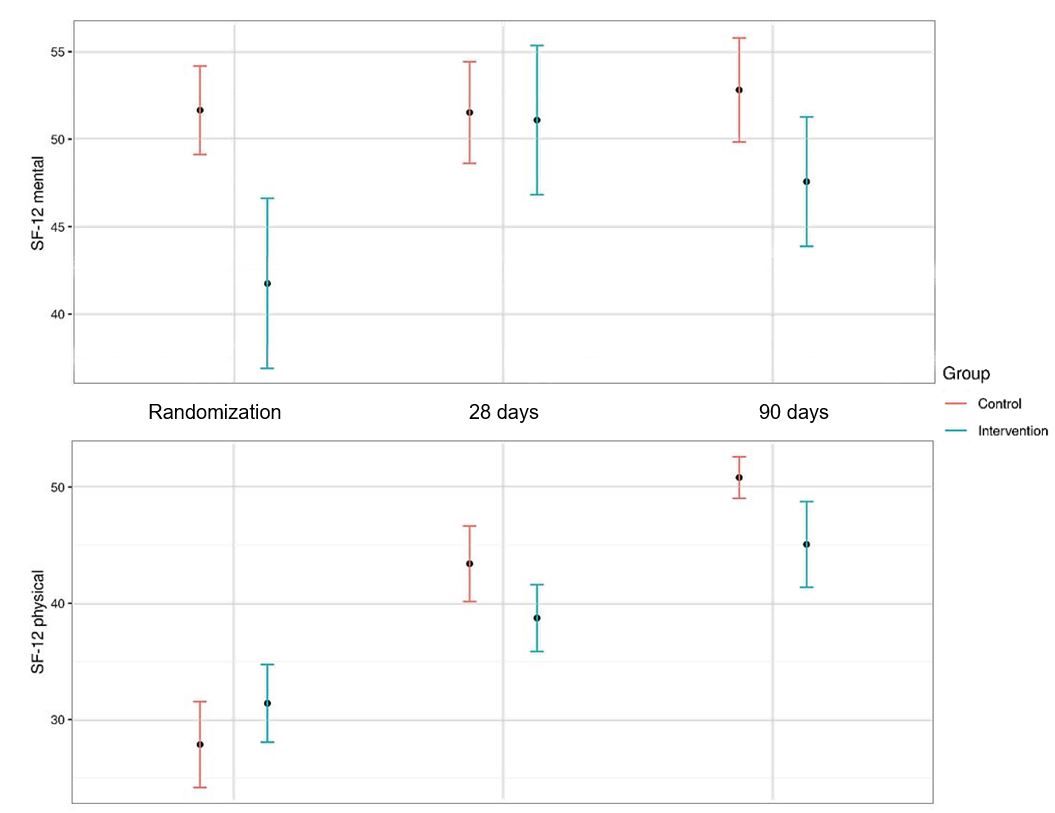


**Supplementary Figure A1**. Group differences in changes in SF-12 mental (top) and SF-12 physical (bottom) across time (randomization =0m, 28 days=1m, 3 months=3m). The mean and 95% confidence interval for the observed mean values are displayed.
